# Supplementary material for: Prediction of 72-hour mortality in patients with extremely high serum C-reactive protein levels using a novel weighted average of risk scores
Source: PLoS One. 2021 Feb 19;16(2):e0246259. doi: 10.1371/journal.pone.0246259 (PMC7894915; doi:10.1371/journal.pone.0246259)
Supplement: S1 Table — ACEI: Angiotensin-converting-enzyme inhibitor, ARB: Angiotensin II receptor blocker, CCB: Calcium channel blocker, NSAID: Nonsteroidal anti-inflammatory agent, PPI: Proton pump inhibitors, HAART: Highly active antiretroviral therapy. P-values were calculated using Fisher’s exact test. (DOCX) [file pone.0246259.s001.docx]

| **S1 Table.** **Clinical profile of patients in both study groups** | | | | | | | | | | |
| --- | --- | --- | --- | --- | --- | --- | --- | --- | --- | --- |
| **Variables** | **Unit** | ***N*** | **72-Hour outcome** | | | | | | | ***P*-value by M-W** |
|  |  |  | **Dead (*n* = 44)**  **Me** (IQR) [*n*] | | | | **Alive (*n* = 231)**  **Me** (IQR) [*n*] | | |  |
| Males, proportion | % | 185 | 70.5 [31] | | | | 66.7 [154] | | | 0.616 |
| Height | cm | 265 | 161.2 (154.6–168.3) [37] | | | | 162.0 (156.0–168.2) [228] | | | 0.616 |
| Weight | kg | 262 | 57.1 (48.2–66.6) [38] | | | | 59.3 (50.1–68.8) [224] | | | 0.314 |
| Body mass index | kg/m^2^ | 258 | 22.6 (19.3–23.5) [36] | | | | 22.5 (19.6–23.4) [222] | | | 0.332 |
| Brinkman index | cigarettes/day × years | 274 | 190 (0–690) [44] | | | | 320 (0–840) [230] | | | 0.326 |
| Vital signs |  |  | | | | | | | | |
| Systolic blood pressure | mmHg | 271 | 110.0 (87.8–122.8) [43] | | | | 116.0 (101.0–130.0) [228] | | | 0.052 |
| Diastolic blood pressure | mmHg | 270 | 65.0 (53.0–72.0) [42] | | | | 66.5 (58.0–78.0) [228] | | | 0.122 |
| Pulse rate | beats/minute | 271 | 109.0 (94.3–123.5) [43] | | | | 103.0 (89.0–120.0) [228] | | | 0.325 |
| Respiratory rate | breath/minute | 177 | 23.5 (17.0–30.0) [30] | | | | 22.0 (18.0–28.0) [147] | | | 0.748 |
| Body temperature | °C | 259 | 37.1 (36.6–38.0) [42] | | | | 37.2 (36.8–38.1) [217] | | | 0.231 |
| Biochemical examinations, excluding the variables listed in Table 1 | | | | | | | | | | |
| WBC | 10^3^/μL | 275 | 13.2 (5.1–18.2) [44] | | | | 12.7 (7.5–19.0) [231] | | | 0.506 |
| Hematocrit | % | 275 | 32.5 (27.4–37.5) [44] | | | | 33.3 (28.4–38.2) [231] | | | 0.520 |
| Platelet count | 10^4^/μL | 275 | 20.7 (8.1–31.9) [44] | | | | 19.9 (11.6–27.7) [231] | | | 0.937 |
| C-reactive protein | mg/dL | 275 | 44.1 (42.0–47.9) [44] | | | | 43.7 (42.0–47.3) [231] | | | 0.905 |
| Total bilirubin | mg/dL | 267 | 0.72 (0.53–1.25) [44] | | | | 0.78 (0.46–1.15) [223] | | | 0.617 |
| Direct bilirubin | mg/dL | 267 | 0.41 (0.29–0.80) [44] | | | | 0.39 (0.23–0.668) [223] | | | 0.169 |
| ALT | U/L | 274 | 25.5 (17.0–41.5) [44] | | | | 23.0 (15.0–45.0) [230] | | | 0.325 |
| γ-Glutamyl transferase | U/L | 226 | 35.0 (20.0–63.3) [37] | | | | 44.0 (22.8–93.5) [189] | | | 0.185 |
| Alkaline phosphatase | U/L | 249 | 256.0 (188.0–400.0) [38] | | | | 258.0 (187.0–382.8) [211] | | | 0.973 |
| CK | U/L | 201 | 156.0 (75.5–830.5) [31] | | | | 211.0 (44.0–704.0) [170] | | | 0.532 |
| Amylase | U/L | 99 | 35.0 (21.0–54.5) [13] | | | | 43.0 (28.0–110.0) [86] | | | 0.147 |
| Na | mEq/L | 273 | 139.0 (134.0–143.5) [44] | | | | 137.0 (134.0–140.0) [229] | | | 0.133 |
| Cl | mEq/L | 273 | 102.0 (97.5–107.5) [44] | | | | 102.0 (97.0–105.0) [229] | | | 0.586 |
| Ca | mEq/L | 219 | 8.00 (7.50–8.40) [38] | | | | 8.00 (7.50–8.63) [181] | | | 0.644 |
| Triglyceride | mg/dL | 72 | 90.0 (55.0–149.0) [8] | | | | 131.0 (92.5–169.5) [64] | | | 0.113 |
| PT-INR |  | 175 | 1.26 (1.18–1.45) [27] | | | | 1.22 (1.25–1.37) [148] | | | 0.156 |
| APTT | seconds | 172 | 44.3 (37.5–54.5) [26] | | | | 41.6 (37.2–48.0) [146] | | | 0.494 |
| AT3 | % | 49 | 48.0 (45.3–51.5) [9] | | | | 67.0 (53.5–75.5) [40] | | | 0.007 |
| Total updated CCI | points | 275 | 2.5 (0.0–9.4) [44] | | | | 2.0 (0.0–8.0) [231] | | | 0.127 |
| **Components of updated CCI (points) 　 　　　N** | | | **Yes, *n* (%)** | | **No, *n* (%)** | | **Yes, *n* (%)** | | **No, *n* (%)** | ***P*-value by Fisher’s exact test** |
| Congestive heart failure (2) | | 275 | 9 (20.5) | | 35 (79.5) | | 37 (16.0) | | 194 (84.0) | 0.470 |
| Dementia (2) | | 275 | 5 (11.4) | | 39 (88.6) | | 1 (0.4) | | 230 (99.6) | 0.000 |
| Chronic pulmonary disease (1) | | 275 | 4 (9.1) | | 40 (90.9) | | 19 (8.2) | 212 (91.8) | | 0.771 |
| Rheumatologic disease (1) | | 275 | 1 (2.3) | | 43 (97.7) | | 15 (6.5) | 216 (93.5) | | 0.482 |
| Mild liver disease (2) | | 275 | 3 (6.8) | 41 (93.2) | | | 22 (9.5) | 209 (90.5) | | 0.777 |
| Hemiplegia or paraplegia (2) | | 275 | 4 (9.1) | 40 (90.9) | | | 15 (6.5) | 216 (93.5) | | 0.519 |
| Mild to severe renal disease (1) | | 275 | 8 (18.2) | 36 (81.8) | | | 58 (25.1) | 173 (74.9) | | 0.324 |
| Diabetes with chronic complications or history of DKA (1) | | 275 | 8 (18.2) | 36 (81.8) | | | 42 (18.2) | 189 (81.8) | | 1.000 |
| Any malignancy, including leukemia and lymphoma (2) | | 275 | 14 (31.2) | 30 (68.2) | | | 53 (22.9) | 178 (77.1) | | 0.021 |
| Moderate or severe liver disease (4) | | 275 | 3 (6.8) | 41 (93.2) | | | 9 (3.9) | 222 (96.1) | | 0.415 |
| Metastatic solid tumor (6) | | 275 | 8 (18.2) | 36 (81.8) | | | 37 (16.2) | 194 (84.0) | | 0.722 |
| Acquired immunodeficiency syndrome/HIV infection (4) | | 275 | 0 (0.0) | 44 (100.0) | | | 1 (0.4) | 230 (99.6) | | 1.000 |
| **Underlying causes N** | | | **Yes, *n* (%)** | | **No, *n* (%)** | | **Yes, *n* (%)** | | **No, *n* (%)** |  |
| Sepsis | | 275 | 8 (18.2) | | 36 (81.8) | | 43 (18.6) | | 188 (81.4) | 0.058 |
| Pneumonia | | 275 | 6 (13.6) | | 38 (86.4) | | 34 (14.7) | 197 (85.3) | | 0.852 |
| Abscess | | 275 | 1 (2.3) | | 43 (97.7) | | 9 (3.9) | 222 (96.1) | | 1.000 |
| Peritonitis | | 275 | 1 (2.3) | | 43 (97.7) | | 4 (1.7) | 227 (98.3) | | 0.116 |
| Gastrointestinal perforation | | 275 | 0 (0.0) | | 44 (100.0) | | 14 (6.1) | 217 (93.9) | | 0.136 |
| Other infections | | 275 | 5 (11.4) | | 39 (88.6) | | 26 (11.3) | 205 (88.7) | | 1.000 |
| Malignancies | | 275 | 10 (22.7) | | 34 (77.3) | | 47 (20.4) | 184 (79.7) | | 0.721 |
| Others | | 275 | 2 (4.5) | | 42 (95.5) | | 35 (15.2) | 196 (84.8) | | 0.002 |
| **Medications　　　　　　　　 N** | | | **Yes, *n* (%)** | | **No, *n* (%)** | | **Yes, *n* (%)** | | **No, *n* (%)** |  |
| Alpha 1-blockers | | 275 | 0 (0.0) | | 44 (100.0) | | 11 (4.8) | | 220 (95.2) | 0.222 |
| Angiotensin converting enzyme inhibitors | | 275 | 0 (0.0) | | 44 (100.0) | | 15 (6.5) | 216 (93.5) | | 0.140 |
| Angiotensin II receptor blockers | | 275 | 3 (6.8) | | 41 (93.2) | | 38 (16.5) | 193 (83.5) | | 0.100 |
| Anti-arrhythmic drugs | | 275 | 2 (4.5) | | 42 (95.5) | | 12 (5.2) | 219 (94.8) | | 1.000 |
| Antibiotics | | 275 | 4 (9.1) | | 40 (90.9) | | 34 (14.7) | 197 (85.3) | | 0.321 |
| Anticoagulants | | 275 | 1 (2.3) | | 43 (97.7) | | 10 (4.3) | 221 (95.7) | | 1.000 |
| Anti-neoplastic drugs | | 275 | 0 (0.0) | | 44 (100.0) |  | 8 (3.5) | 223 (96.5) | | 0.363 |
| Antiplatelets | | 275 | 8 (18.2) | | 36 (81.8) | | 46 (19.9) | 185 (80.1) | | 0.791 |
| Antipsychotics | | 275 | 3 (6.8) | | 41 (93.2) | | 27 (11.7) | 204 (88.3) | | 0.437 |
| Antiseizure drugs | | 275 | 0 (0.0) | | 44 (100.0) | | 8 (3.5) | 223 (96.5) | | 0.363 |
| Beta-blockers | | 275 | 4 (9.1) | | 40 (90.9) | | 25 (10.8) | 206 (89.2) | | 1.000 |
| Bisphosphonates | | 275 | 1 (2.3) | | 43 (97.7) | | 1 (0.4) | 230 (99.6) | | 0.295 |
| Calcium channel blockers | | 275 | 7 (15.9) | | 37 (84.1) | | 48 (20.8) | 183 (79.2) | | 0.459 |
| Cholinesterase inhibitors for dementia | | 275 | 2 (4.5) | | 42 (95.5) | | 0 (0.0) | 231 (100.0) | | 0.025 |
| Diuretics | | 275 | 4 (9.1) | | 40 (90.9) | | 32 (13.9) | 199 (86.1) | | 0.390 |
| H_2_-blockers | | 275 | 3 (6.8) | | 41 (93.2) | | 34 (14.7) | 197 (85.3) | | 0.159 |
| HAART | | 275 | 0 (0.0) | | 44 (100.0) | | 2 (0.9) | 229 (99.1) | | 1.000 |
| Hypnotics | | 275 | 5 (11.4) | | 39 (88.6) | | 27 (11.7) | 204 (88.3) | | 0.951 |
| Hypoglycemic agents | | 275 | 4 (9.1) | | 40 (90.9) | | 34 (14.7) | 197 (85.3) | | 0.321 |
| Immunosuppressants | | 275 | 2 (4.5) | | 42 (95.5) | | 13 (5.6) | 218 (94.4) | | 1.000 |
| NSAIDs | | 275 | 5 (11.4) | | 39 (88.6) | | 38 (16.5) | 193 (83.5) | | 0.394 |
| Opioids | | 275 | 6 (13.6) | | 38 (86.4) | | 18 (7.8) | 213 (92.2) | | 0.240 |
| Proton pump inhibitors | | 275 | 8 (18.2) | | 36 (81.8) | | 65 (28.1) | 166 (71.9) | | 0.170 |
| Statins | | 275 | 4 (9.1) | | 40 (90.9) | | 36 (15.6) | 195 (84.4) | | 0.263 |
| Steroids | | 275 | 2 (4.5) | | 42 (95.5) | | 26 (11.3) | 205 (88.7) | | 0.275 |
| Urate-lowering drugs | | 275 | 3 (6.8) | | 41 (93.2) | | 29 (12.6) | 202 (87.4) | | 0.277 |
| Others | | 275 | 20 (45.5) | | 24 (54.5) | | 141 (61.0) | 90 (39.0) | | 0.054 |

Abbreviations: HIV: Human immunodeficiency virus, DKA: Diabetic ketoacidosis, HHS: Hyperosmolar hyperglycemic state, ACS: Acute coronary syndrome, AHF: Acute heart failure, IQR: Inter-quartile range.

ACEI: Angiotensin-converting-enzyme inhibitors, ARB: Angiotensin II receptor blockers, CCB: Calcium channel blockers, NSAID: Nonsteroidal anti-inflammatory agents, PPI: Proton pump inhibitors, HAART: Highly active antiretroviral therapy.

*P*-values were calculated using Fisher’s exact test for nominal variables and the Mann–Whitney *U* test for numerical variables*.*
