# Supplementary material for: Prediction of 72-hour mortality in patients with extremely high serum C-reactive protein levels using a novel weighted average of risk scores
Source: PLoS One. 2021 Feb 19;16(2):e0246259. doi: 10.1371/journal.pone.0246259 (PMC7894915; doi:10.1371/journal.pone.0246259)

PLOS ONE

Predicting 72-hour mortality in patients with extremely high serum C-reactive protein levels over 40 mg/dL: a case-controlled cross-sectional study.

--Manuscript Draft--

**Manuscript Number:**	PONE-D-20-07042
**Article Type:**	Research Article
**Full Title:**	Predicting 72-hour mortality in patients with extremely high serum C-reactive protein levels over 40 mg/dL: a case-controlled cross-sectional study.
**Short Title:**	Predicting 72-hour mortality in patients with extremely high serum C-reactive protein levels
**Corresponding Author:**	Hitoshi Sugawara, MD, PhD, FACP<br>Jichi Medical University<br>Saitama, Saitama JAPAN
**Keywords:**	C-reactive protein; 72-hour mortality prediction; extreme outlier value; panic data; logistic regression analysis; serum inorganic phosphorus; blood urea nitrogen
**Abstract:**	The risk factors associated with 72-hour mortality in patients with extremely high serum C-reactive protein (CRP) levels remain unclear. We aim to explore the predictive factors of 72-hour mortality in patients with extremely high levels of CRP. In this retrospective single-center case-controlled cross-sectional study, data were obtained from the medical records of patients ≥18 years old in the Saitama Medical Center, Japan, from 2004 to 2013. The primary outcome was defined as 72-hour mortality following the CRP test, regardless of whether the patients were hospitalized or in an outpatient setting. Multivariate logistic regression analysis was performed with adjustment for age, sex, and CRP level. The final prediction model was built using the logistic regression model with higher C-statistic, specificity, and sensitivity. A total of 183 patients with CRP levels over 40 mg/dL were identified over 10 years. The 72-hour mortality rate was 26/183 (14.2%), and the C-statistic of the 72-hour mortality prediction model with age, serum inorganic phosphorus, and blood urea nitrogen (BUN) were 0.868. The probability of death, i.e., the 72-hour mortality, was as follows: 0.114 [age] + 2.759 [serum inorganic phosphorus] – 1.616 × [BUN] – 7.72. The sensitivity and specificity of this model was 72.0%. In patients with CRP levels over 40 mg/dL, the main independent risk factors associated with 72-hour mortality were age, elevated serum inorganic phosphorus, and elevated BUN. Further studies are required to understand the mechanisms and possible interventions to prevent death associated with extremely high serum CRP levels.
**Order of Authors:**	Kai Saito
	Hitoshi Sugawara, MD, PhD, FACP
	Tamami Watanabe
	Akira Ishii
	Takahiko Fukuchi
**Additional Information:**	
**Question**	**Response**
**Financial Disclosure**<br><br>Enter a financial disclosure statement that describes the sources of funding for the work included in this submission. Review the submission guidelines for detailed requirements. View published research articles from *PLOS ONE* for specific examples.	This work was supported by JSPS KAKENHI (Grant Number: 26460916). HS received this grant award. The funders had no role in study design, data collection and analysis, decision to publish, or preparation of the manuscript.

Powered by Editorial Manager® and ProduXion Manager® from Aries Systems Corporation

This statement is required for submission and **will appear in the published article** if the submission is accepted. Please make sure it is accurate.<br><br>**Unfunded studies**<br>Enter: *The author(s) received no specific funding for this work.*<br><br>**Funded studies**<br>Enter a statement with the following details:<br>• Initials of the authors who received each award<br>• Grant numbers awarded to each author<br>• The full name of each funder<br>• URL of each funder website<br>• Did the sponsors or funders play any role in the study design, data collection and analysis, decision to publish, or preparation of the manuscript?<br>• **NO** - Include this sentence at the end of your statement: *The funders had no role in study design, data collection and analysis, decision to publish, or preparation of the manuscript.*<br>• **YES** - Specify the role(s) played.<br><br>* typeset	
**Competing Interests**<br><br>Use the instructions below to enter a competing interest statement for this submission. On behalf of all authors, disclose any competing interests that could be perceived to bias this work—acknowledging all financial support and any other relevant financial or non-financial competing interests.<br><br>This statement **will appear in the published article** if the submission is accepted. Please make sure it is accurate. View published research articles from *PLOS ONE* for specific examples.	The authors have declared that no competing interests exist.

Powered by Editorial Manager® and ProduXion Manager® from Aries Systems Corporation

**NO authors have competing interests**<br><br>Enter: *The authors have declared that no competing interests exist.*<br><br>**Authors with competing interests**<br><br>Enter competing interest details beginning with this statement:<br><br>*I have read the journal's policy and the authors of this manuscript have the following competing interests: [insert competing interests here]*<br><br>* typeset	
**Ethics Statement**<br><br>Enter an ethics statement for this submission. This statement is required if the study involved:<br><br>• Human participants<br>• Human specimens or tissue<br>• Vertebrate animals or cephalopods<br>• Vertebrate embryos or tissues<br>• Field research<br><br>Write "N/A" if the submission does not require an ethics statement.<br><br>General guidance is provided below. Consult the submission guidelines for detailed instructions. **Make sure that all information entered here is included in the Methods section of the manuscript.**	The study protocol was approved by the Institutional Clinical Research Ethics Review Board of Saitama Medical Center, Jichi Medical University, Saitama, Japan (Clinical #10-79). Because of the retrospective nature of the study, the requirement for informed consent was waived.

Powered by Editorial Manager® and ProduXion Manager® from Aries Systems Corporation

**Format for specific study types**<br><br>**Human Subject Research (involving human participants and/or tissue)**<br>• Give the name of the institutional review board or ethics committee that approved the study<br>• Include the approval number and/or a statement indicating approval of this research<br>• Indicate the form of consent obtained (written/oral) or the reason that consent was not obtained (e.g. the data were analyzed anonymously)<br><br>**Animal Research (involving vertebrate animals, embryos or tissues)**<br>• Provide the name of the Institutional Animal Care and Use Committee (IACUC) or other relevant ethics board that reviewed the study protocol, and indicate whether they approved this research or granted a formal waiver of ethical approval<br>• Include an approval number if one was obtained<br>• If the study involved *non-human primates*, add *additional details* about animal welfare and steps taken to ameliorate suffering<br>• If anesthesia, euthanasia, or any kind of animal sacrifice is part of the study, include briefly which substances and/or methods were applied<br><br>**Field Research**<br><br>Include the following details if this study involves the collection of plant, animal, or other materials from a natural setting:<br>• Field permit number<br>• Name of the institution or relevant body that granted permission	
**Data Availability**<br><br>Authors are required to make all data underlying the findings described fully available, without restriction, and from the time of publication. PLOS allows rare exceptions to address legal and ethical concerns. See the PLOS Data Policy and FAQ for detailed information.	No - some restrictions will apply

A Data Availability Statement describing where the data can be found is required at submission. Your answers to this question constitute the Data Availability Statement and **will be published in the article**, if accepted.<br><br>**Important:** Stating 'data available on request from the author' is not sufficient. If your data are only available upon request, select 'No' for the first question and explain your exceptional situation in the text box.<br><br>Do the authors confirm that all data underlying the findings described in their manuscript are fully available without restriction?	
**Describe where the data may be found in full sentences. If you are copying our sample text, replace any instances of XXX with the appropriate details.**<br><br>• If the data are **held or will be held in a public repository**, include URLs, accession numbers or DOIs. If this information will only be available after acceptance, indicate this by ticking the box below. For example: *All XXX files are available from the XXX database (accession number(s) XXX, XXX.).*<br>• If the data are all contained **within the manuscript and/or Supporting Information files**, enter the following: *All relevant data are within the manuscript and its Supporting Information files.*<br>• If neither of these applies but you are able to provide **details of access elsewhere**, with or without limitations, please do so. For example:<br><br>*Data cannot be shared publicly because of [XXX]. Data are available from the XXX Institutional Data Access / Ethics Committee (contact via XXX) for researchers who meet the criteria for access to confidential data.*<br><br>*The data underlying the results presented in the study are available from (include the name of the third party*	The data are available to interested researchers upon reasonable request to the corresponding author, pending ethical approval.

and contact information or URL).<br>• This text is appropriate if the data are owned by a third party and authors do not have permission to share the data.<br><br>* typeset	
Additional data availability information:	

Manuscript Click here to download Manuscript Manuscript.doc

Predicting 72-hour mortality in patients with extremely high serum C-reactive protein levels over 40 mg/dL: a case-controlled cross-sectional study

Kai Saito[1], Hitoshi Sugawara[2*], Tamami Watanabe[2], Akira Ishii[2], Takahiko Fukuchi[2]

[1]Nara Medical University, Nara, Japan,

[2]Division of General Medicine, Department of Comprehensive Medicine 1, Saitama Medical Center, Jichi Medical University, Saitama, Japan

Short title: Predicting 72-hour mortality in patients with extremely high serum C-reactive protein levels

Corresponding author:

E-mail: hsmdfacp@jichi.ac.jp (HS)

Abstract

The risk factors associated with 72-hour mortality in patients with extremely high serum C-reactive protein (CRP) levels remain unclear. We aim to explore the predictive factors of 72-hour mortality in patients with extremely high levels of CRP. In this retrospective single-center case-controlled cross-sectional study, data were obtained from the medical records of patients ≥18 years old in the Saitama Medical Center, Japan, from 2004 to 2013. The primary outcome was defined as 72-hour mortality following the CRP test, regardless of whether the patients were hospitalized or in an outpatient setting. Multivariate logistic regression analysis was performed with adjustment for age, sex, and CRP level. The final prediction model was built using the logistic regression model with higher C-statistic, specificity, and sensitivity. A total of 183 patients with CRP levels over 40 mg/dL were identified over 10 years. The 72-hour mortality rate was 26/183 (14.2%), and the C-statistic of the 72-hour mortality prediction model with age, serum inorganic phosphorus, and blood urea nitrogen (BUN) were 0.868. The probability of death, i.e., the 72-hour mortality, was as follows: 0.114 [age] + 2.759 [serum inorganic phosphorus] – 1.616 × [BUN] – 7.72. The sensitivity and specificity of this model was 72.0%. In patients with CRP levels over 40 mg/dL, the main independent risk factors associated with 72-hour mortality were age, elevated serum inorganic phosphorus, and elevated BUN. Further studies are required to understand the mechanisms and possible interventions to prevent death associated with extremely high serum CRP levels.

Keywords: C-reactive protein, 72-hour mortality prediction, extreme outlier value, panic data, logistic regression analysis, serum inorganic phosphorus, blood urea nitrogen.

Introduction

Clinical data accumulate at a high rate in daily practice at medical institutions. Generally, clinical data are used to generate alerts to improve the clinical practice and ensure that the most appropriate care is provided to the patient. Reuse or secondary use of clinical data is an emerging field that is recognized as being essential for delivering high-quality healthcare and improved healthcare management [1, 2]. By examining the clinical data utility, presenters considered how the structure of the medical care data marketplace can affect research priorities, gaps, and possibilities. Health information technology may have the potential to improve the collection and exchange of personal health records allowing for utilization in electronic form [1, 2]. However, reuse or secondary use of clinical data for the improvement of the overall quality of medical care remains limited.

Critical values represent a pathophysiological state at such variance with the reference values. Critical values may constitute an immediate health risk to the individual and become life-threatening without prompt action. One such example is serum potassium (K) levels of 2.6 mmol/L [3]. Meanwhile, extreme outlier values are those that lie statistically outside the 0.1–99.0 percentile rang [4]. Precisely, these critical values have gained interest in the efficacy of identifying and communicating relevant information to treating physicians [5]. Therefore, physicians who encounter patients with extreme outlier laboratory values may be unsure if such values are critical or not.

C-reactive protein (CRP), a phylogenetically highly conserved plasma protein, is an acute inflammatory protein primarily synthesized in hepatocytes [6], smooth muscle cells, macrophages, endothelial cells, lymphocytes, and adipocytes [7]. CRP plays versatile roles in

either physiological or pathophysiological states [8], and the plasma concentration of CRP increases during systemic inflammatory responses, a characteristic that has long been employed for clinical purposes and used as a biomarker for acute inflammation. CRP rapidly increases within hours of tissue injury or infection and participates in host defense as a part of the innate immune response [9]. Recent studies have shown that CRP levels are related to the prognosis or disease activity of various diseases [10], such as acute ischemic stroke [11], urological cancer [12], advanced non-small cell lung cancer [13], acute idiopathic pericarditis [14], the onset of immune-related adverse events in patients treated with immune checkpoint inhibitors in the absence of an infectious disease [15], heart transplant patients [16], patients hospitalized in internal medicine wards [17], as well as being an acute care coronary heart disease risk factor [18].

The median concentration of CRP in healthy young adults is 0.08 mg/dL, the 90th centile is 0.3 mg/dL, and the 99th is 1.0 mg/dL [9, 19, 20]. In hospital settings, physicians may encounter patients with CRP levels that significantly exceed the normal range, from less than 50 µg/l to more than 500 mg/l, that is, 10,000-fold change [9, 19, 20]. An acute stimulus very rapidly initiates *de novo* synthesis of CRP in hepatocytes, rises above 5 mg/l within 4–8 hours, and peaks at 48 hours. The half-life of plasma CRP is 19 hours and is constant in all conditions of health and disease [9, 19, 20]. However, the prediction model of 72-hour mortality for patients with an extremely high outlier value of CRP remains elusive.

Hence, the purpose of this retrospective study is to determine the association between very-early mortality and predictive factors in the 72 hours following the test date of patients who demonstrate extremely high outlier values of CRP. Predicting very-early mortality within 72 hours is extremely important for both primary and critical care settings. We hypothesized that the

creation of a novel 72-hour prognosis prediction model for use in patients with extremely high CRP levels could help physicians in decision making and provide therapeutic and management options to patients and their families. We anticipate an improvement in the quality of initial medical management because of implementing these changes.

Methods

The study protocol was designed according to the tenets of the Declaration of Helsinki [21] and was approved by the Institutional Clinical Research Ethics Review Board of Saitama Medical Center, Jichi Medical University, Saitama, Japan (Clinical #10-79). Because of the retrospective nature of the study, the requirement for informed consent was waived.

Study design and participant selection

The present study was a retrospective single-center case-controlled cross-sectional study. We used the descending order of the CRP data list (displaying all 908,750 patients) gleaned at the clinical laboratory of our medical center from 2004 to 2013, and extracted 283 patients aged over 18 by setting the extreme high outlier value of CRP over 40 mg/dL [22] in which the occurrence rate was 0.1%. We excluded patients that were metachronous duplicates from the same patient (only one highest value of CRP from each of these patients was considered), patients with a cardiopulmonary arrest at arrival, and patients with unknown outcomes. After applying the exclusion criteria, a sample of 167 patients was selected for further analysis. A flow chart of the selected cohort is shown in Fig 1.

Fig 1. Flow diagram outlining the patient selection

When setting the extreme high outlier value of CRP over 40 mg/dL, 281 patients were selected from the CRP data list that included 908,750 patients from 2004 to 2013 who visited our medical center. CRP values were arranged in descending order at time of selection. The exclusions included 10 patients with cardiopulmonary arrest at arrival, 5 patients with unknown outcomes, and 83 patients that were metachronously duplicates from the same patient (only the highest CRP value was taken from each of these patients). Finally, 183 patients were enrolled in this study. The 72-hour outcomes were 26 deaths and 157 survivals.

The primary outcome was defined as 72-hour mortality [23, 24] following the CRP test, regardless of whether the patient was hospitalized or in an outpatient setting. Cases were defined as patients with an extremely high level of CRP who died in hospital during the first 72 hours after the test, while the controls were patients with an extremely high level of CRP who survived.

The following risk factors were tested for their association with the 72-hour mortality outcome: age, sex, height, weight, body mass index (BMI), number of cigarettes smoked (Brinkman index), vital signs at the time of examination (e.g., systolic blood pressure, diastolic blood pressure, heart rate, respiratory rate, and body temperature), laboratory test values (e.g., total protein, albumin, total bilirubin, direct bilirubin, aspartate transaminase [AST], alanine transaminase [ALT], γ-glutamyl transpeptidase [γ-GTP], lactate dehydrogenase [LD], alkaline phosphatase [ALP], creatine kinase [CK], amylase, C-reactive protein [CRP], sodium [Na], potassium [K], chloride [Cl], calcium [Ca], inorganic phosphorus [P], blood urea nitrogen [BUN], creatinine [Cr], uric acid, total cholesterol, and random plasma glucose), as well as total updated Charlson comorbidity index (CCI) scores [25], CCI components, and underlying causes of extremely high CRP.

Statistical analysis

Sample size

We calculated the required sample size as 23 deaths and 138 survivals to achieve a power of 80% and an alpha error of 5%, assuming an odds ratio of 4.0 for risk factors related to 72-hour mortality [26].

Descriptive statistics

All continuous data were checked for the assumption of normality. All variables are presented as the median and the 2.5–97.5 percentiles. If the volume of data was insufficient to calculate the 2.5–97.5 percentile, only median values were displayed. Variables with a skewed distribution were logarithmically transformed before regression analyses [27]. The group differences were tested using Fisher's exact test for nominal variables and the Mann-Whitney U-test for continuous variables.

Association analysis

The primary focus was to determine multiple risk factors associated with 72-hour mortality. First, the potential risk factors for 72-hour mortality were examined using univariate logistic regression analysis. The odds ratios (OR) and 95% confidence interval (CI) were estimated without adjustment (crude ORs). For covariates with a significant crude OR, we determined the C-statistics by receiver-operating characteristic (ROC) analysis. Second, we performed multivariable logistic regression analysis. We estimated the OR with 95% CI and adjusted for age, sex, and CRP level for all covariates that were significant in the univariate

logistic regression analysis and the C-statistics by the ROC analysis. Clinically important risk factors that were significant in the logistic regression analysis were included in the final prediction model. The final prediction model was estimated using multivariate logistic regression analysis with a stepwise model building algorithm. Stepwise regression for all continuous data is a method of regressing multiple variables while simultaneously removing those that are not considered important, so the selected model includes the optimal combination of covariates of continuous data. In the final step, binary covariates, such as CCI components and causes of extremely high CRP, were added to the former selected model with continuous data and analyzed using multivariate logistic regression analysis with a stepwise model building algorithm. In the final prediction model, the logit function reflected the probability of death, defined as the 72-hour mortality.

The following statistics were estimated in multivariate logistic regression and ROC analyses: intercept, regression coefficient, $\chi 2$ value, p-value, variance inflation factor (VIF), adjusted OR, 95% CI, C-statistics, sensitivity, and specificity. A p-value of < 0.05 was accepted as statistically significant. C-statistics was defined as the area under the curve (AUC) analyzed according to the ROC curve. In addition, we assessed the multiple collinearities between covariates by the VIF. Multiple collinearities were defined as positive when the VIF was 5 or greater.

Statistical software

The statistical package for the StatFlex software version 7.0.10 (Artech Co. Ltd, Osaka, Japan) was used for data analysis. G*Power version 3.1.9.4 [26] was used for sample size calculation.

Results

Participant demographics

The characteristics of the study groups, such as patient demographics, vital signs, laboratory test values, updated CCI, and underlying causes of extremely high CRP, are presented in Tables 1 and 2. The 72-hour mortality rate was 26/183 (14.2%), and the survival rate was 157/183 (85.8%). There was no significant difference in the CRP levels between the deceased (44.4 mg/dL) and survival (44.8 mg/dL) groups. The demographic data, age, and biochemical data, such as potassium, inorganic phosphorus, and BUN, were significantly higher in the death groups than the survival groups. Conversely, albumin, and total cholesterol were significantly higher in the survival control groups than the death groups (Table 1). Other demographic data, vital signs, and biochemical data showed no significant differences between the death and survival groups.

Table 1. Patient demographics, vital signs, laboratory test values, and updated Charlson comorbidity index

	Very-early mortality				
	Death (n = 26)		Survival (n = 157)		*P*-value
	Median (2.5–97.5 percentile)	Number	Median (2.5–97.5 percentile)	Number	
Age (years)	72.0 (31.3–92.7)	26	64.0 (33.4–84.0)	157	0.013
Sex (men)		19 (73.1%)		104 (66.2%)	0.492
Height (cm)	162.1 (144.6–175.0)	20	162.0 (144.3–178.8)	153	0.934
Weight (kg)	55.0 (45.0–88.5)	22	58.6 (39.0–87.9)	155	0.572
Body mass index (kg/m^2)	21.0 (15.2–30.8)	20	22.2 (15.2–31.5)	153	0.458
Brinkman index	420 (0–1,500)	20	442 (0–2,390)	142	0.804
Vital signs					
Systolic blood pressure (mmHg)	110.0 (51.0–166.4)	22	114.0 (72.2–160.0)	122	0.530

Diastolic blood pressure (mmHg)	68.0 (29.3–119.5)	21	69.5 (44.1–101.8)	122	0.773
Pulse rate (beats/minute)	112.5 (72.4–150.0)	22	106.0 (60.1–154.2)	121	0.288
Respiratory rate (breath/minute)	29.0 (7.0–43.0)	12	24.0 (12.0–48.0)	61	0.655
Body temperature (°C)	37.4 (35.4–38.3)	21	37.4 (35.8–39.8)	115	0.412
Biochemical examinations					
Total protein (g/dL)	5.60 (4.31–7.29)	23	5.80 (4.20–7.96)	148	0.311
Albumin (g/dL)	2.10 (0.93–3.84)	23	2.50 (1.32–3.60)	148	0.026
Total bilirubin (mg/dL)	0.70 (0.25–4.90)	25	0.80 (0.27–4.97)	147	0.824
Direct bilirubin (mg/dL)	0.39 (0.14–3.12)	25	0.40 (0.11–4.03)	147	0.539
Aspartate transaminase (U/L)	33.0 (13.2–2,474.3)	26	35.0 (9.8–667.9)	155	0.779
Alanine transaminase (U/L)	23.5 (5.3–1,328.5)	26	25.0 (5.4–335.3)	155	0.530
γ-Glutamyl transferase (U/L)	34.0 (4.2–172.0)	21	46.5 (9.0–441.5)	122	0.148

Lactate dehydrogenase (U/L)	253.0 (139.0–6,667.4)	24	292.0 (121.5–3,059.9)	147	0.745
Alkaline phosphatase (U/L)	247.5 (106.0–712.0)	20	268.5 (127.6–1,372.7)	136	0.383
Creatine kinase (U/L)	111.0 (12.0–31,402.0)	15	216.0 (8.4–17,188.0)	108	0.536
Amylase (U/L)	28.5 (4.0–81.0)	6	42.0 (8.7–1,079.3)	69	0.156
C-reactive protein (mg/dL)	44.4 (40.2–58.2)	26	44.8 (40.3–60.6)	157	0.643
Sodium (mEq/L)	137.5 (128.0–152.4)	22	137.0 (126.0–155.3)	154	0.972
Potassium (mEq/L)	4.35 (2.79–8.84)	26	4.00 (2.61–5.77)	154	0.018
Chloride (mEq/L)	101.5 (80.4–116.8)	26	101.0 (86.7–118.3)	154	0.621
Calcium (mg/dL)	7.95 (4.50–9.40)	14	8.10 (5.10–10.4)	94	0.506
Inorganic phosphorus (mg/dL)	4.85 (1.60–8.70)	14	3.15 (1.20–8.12)	94	0.023
Blood urea nitrogen (mg/dL)	56.0 (9.5–95.9)	25	35.0 (11.1–104.9)	155	0.006
Creatinine (mg/dL)	1.61 (0.49–16.13)	25	1.38 (0.461–8.89)	155	0.572

Uric acid (mg/dL)	7.90 (1.70–17.00)	12	6.05 (2.55–16.56)	78	0.573
Total cholesterol (mg/dL)	102.0 (56.0–132.0)	5	130.0 (81.4–216.6)	45	0.017
Random plasma glucose (mg/dL)	149.5 (60.0–456.0)	6	150.5 (86.1–652.1)	42	0.596
Updated Charlson comorbidity index					
Total score	2.0 (0.0–6.0)	26	2.0 (0.0–8.0)	157	0.793

P-values were calculated using the Mann–Whitney *U*-test.

183 Table 2. Characteristics, patient demographics, Charlson comorbidity index components, and underlying causes

	Very-early mortality		Total	
	Death (n = 26)	Survival (n = 157)		*P*-value
	n (%)	n (%)	n (%)	
Components of updated Charlson comorbidity index (score)				
Congestive heart failure (2)	4 (15.4)	32 (20.4)	36 (19.7)	0.553
Dementia (2)	4 (15.4)	1 (0.6)	5 (2.7)	0.001
Chronic pulmonary disease (1)	3 (11.5)	8 (5.1)	11 (6.0)	0.192
Rheumatologic disease (1)	1 (3.8)	7 (4.5)	8 (4.4)	1.000
Mild liver disease (2)	0 (0.0)	8 (5.1)	8 (4.4)	0.603
Hemiplegia or paraplegia (2)	0 (0.0)	12 (7.6)	12 (6.6)	0.221
Mild to severe renal disease (1)	2 (7.7)	35 (22.3)	37 (20.2)	0.086

Diabetes with chronic complications or history of DKA (1)	2 (7.7)	17 (10.8)	19 (10.4)	1.000
Any malignancy, including leukemia and lymphoma (2)	9 (34.6)	32 (20.4)	41 (22.4)	0.107
Moderate or severe liver disease (4)	1 (3.8)	2 (1.3)	3 (1.6)	0.370
Metastatic solid tumor (6)	4 (15.4)	29 (18.5)	33 (18.0)	1.000
Acquired immunodeficiency syndrome/HIV infection (4)	0 (0.0)	1 (0.6)	1 (0.5)	1.000
Underlying causes of extremely high CRP				
Sepsis	4 (15.4)	37 (23.6)	41 (22.4)	0.354
Pneumonia	5 (19.2)	25 (15.9)	30 (16.4)	0.774
Abscess	1 (3.8)	7 (4.5)	8 (4.4)	1.000
Peritonitis	1 (3.8)	3 (1.9)	4 (2.2)	0.461
Other infectious diseases	2 (7.7)	19 (12.1)	21 (11.5)	0.743
Malignancy	6 (23.1)	26 (16.6)	32 (17.5)	0.410

Vascular disease	4 (15.4)	14 (8.9)	18 (9.8)	0.294
Gastrointestinal perforation	0 (0.0)	5 (3.2)	5 (2.7)	1.000
Others	3 (11.5)	21 (13.4)	24 (13.1)	1.000
Total	26 (100.0)	157 (100.0)	183 (100.0)	

HIV: Human immunodeficiency virus, DKA: Diabetic ketoacidosis, HHS: Hyperosmolar hyperglycemic syndrome, ACS: Acute coronary syndrome, AHF: Acute heart failure

P-values were calculated using Fisher's exact test.

The statistical analysis of CCI components revealed that the proportion of patients with dementia was significantly higher in cases compared to the survival controls (15.4% vs. 0.6%, p = 0.001). There was no statistical significance observed in the other components of CCI. The statistical analysis of the underlying causes of extreme high-CRP showed no significant differences between the groups.

Univariate and multivariate logistic regression analysis

The crude (unadjusted) regression analysis revealed that the significant risk factors associated with increased risk of the primary outcome were age, potassium, inorganic phosphorus, BUN, and dementia of CCI components. Interestingly, the risk factors showed a protective effect only with regards to albumin (Table 3).

198 Table 3. Univariate and multivariate logistic regression analysis results

	Univariate logistic regression analysis			Multivariate logistic regression analysis		
	Crude odds ratio (95% CI)	*P*-value	C-statistics	Adjusted odds ratio (95% CI) by age, sex, and CRP	*P*-value	C-statistics
Characteristics of patients						
Age (year)	1.040 (1.004–1.078)	0.027	0.652	1.038 (1.002–1.076)	0.039	0.661
Components of updated Charlson comorbidity index						
Dementia	28.1 (3.01–263.7)	0.003	0.574	17.66 (1.673–186.3)	0.017	0.657
Biochemical examinations						
Albumin (g/dL)	0.922 (0.855–0.995)	0.037	0.644	0.928 (0.857–1.004)	0.064	0.731
Potassium (mEq/L)	1.347 (1.085–1.672)	0.007	0.645	1.372 (1.095–1.718)	0.006	0.710
Inorganic phosphorus (mg/dL)	1.153 (1.017–1.308)	0.026	0.688	1.145 (0.999–1.316)	0.052	0.811

Blood urea nitrogen (mg/dL)	2.572 (1.160–5.703)	0.020	0.673	2.335 (1.034–5.274)	0.041	0.691

CI: Confidence interval, VIF: Variance inflation factor

P-values were calculated using the $\chi2$ test.

In terms of calculating the odds ratio, the variation of albumin, potassium, and serum inorganic phosphorus was 0.1. The variation of others was 1.0. Remarkably, when adjusted for age, sex, and CRP levels, the most potent risk factor associated with an increased risk of 72-hour mortality was dementia of CCI components. All other risk factors, except albumin and inorganic phosphorus, were insignificant by testing with the univariate model. In addition, the C-statistic of serum inorganic phosphorus was the highest at 0.811 (Table 3).

Prediction model

Three covariates, i.e., age, serum inorganic phosphorus, and BUN, were selected using stepwise regression analysis as the most significant predictors. The best combination of covariates to predict 72-hour mortality with the highest C-statistics (C-statistic, 0.868) was age, serum inorganic phosphorus, and BUN. The final prediction model coefficients were: 0.114 × [age, years] + 2.759 [serum inorganic phosphorus value, mg/dL] – 1.616 [BUN value, mg/dL] - 7.72. This model predicted the 72-hour mortality of patients with an extremely high CRP level, with 72.0% for both sensitivity and specificity (Table 4; Fig 2).

Fig. 2. Receiver-operating characteristic analysis of predictive diagnosis models for the 72-hour mortality of patients with extremely high outlier C-reactive protein values.

The dotted line shows the reference curve for age, sex, and C-reactive protein. The solid line shows the model, including the best combination (age, serum inorganic phosphorus, and blood urea nitrogen), estimated using logistic regression analysis by a stepwise model building algorithm.

Table 4. Comparison of multivariate logistic regression analysis models

Variable selection		β	SE(β)	Z	*P*-value	VIF	Adjusted odds ratio	95% CI	C-statistics	Sn (%)	Sp (%)
Reference	Intercept	-1.02	8.485						0.661	65.4	65.4
	Age	0.037	0.018	2.060	0.039	1.040	1.038	1.002–1.076			
	Sex	0,233	0.483	0.483	0.629	1.014	1.263	0.490–3.255			
	C-reactive protein	-0.812	2.160	-0.413	0.680	1.034	0.915	0.599–1.397			
Model	Intercept	-7.72	3.130						0.868	72.0	72.0
	Age	0.114	0.036	3.152	0.002	1.037	1.121	1.044–1.203			
	Inorganic phosphorus	2.759	1.089	2.533	0.011	1.395	1.318	1.064–1.631			
	Blood urea nitrogen	-1.616	0.791	-2.044	0.041	1.430	0.199	0.042–0.936			

SE: Standard error, CI: Confidence interval, Sn: Sensitivity, Sp: Specificity

The variation of C-reactive protein and serum inorganic phosphorus was 0.1. The variation of the others was 1.0.

P-values were calculated using the χ^2 test.

Discussion

The present study is the first hospital-based study to investigate the risk factors associated with 72-hour mortality in people with extremely high CRP over 40 mg/dL. The main findings were that the 72-hour mortality rate in patients with extremely high CRP was 14.2% and that the main independent risk factors associated with 72-hour mortality were age, serum inorganic phosphorus, and BUN. The average life expectancy decreases and the age-specific mortality inceases by age in adult [28]. Concerning the relation between aging and CRP, a recent report indicate that that cardiovascular risk prediction by N-terminal pro brain natriuretic peptide and high sensitivity CRP was affected by age and sex [29]. In our study, the median (2.5–97.5 percentile) age of the deceased groups was 72.0 (31.3–92.7). In contrast, these of the survival group was 64.0 (33.4–84.0), which was significantly high in the deceased group. The adjusted odds ratio was 1.038, which is lower than both of these of serum inorganic phosphorus and BUN.

CRP is an expedient inflammatory biomarker with rapid kinetics for inflammatory responses. The association between prognosis and high CRP has been reported in several diseases including septic shock [30], bacterial infection [31], acute ischemic stroke [11], acute idiopathic pericarditis [14], unstable angina or non-Q-wave myocardial infarction [32], and most adult solid tumors [33] including advanced non-small cell lung cancer [13], endometrial cancer [34], urothelial cancer along with renal cell carcinoma, prostate cancer, bladder cancer, and upper urinary tract urothelial carcinoma [12]. Furthermore, a recent study demonstrated significant differences in CRP levels between patients who died and those who survived at 48 h and 96 h after admission to the palliative care unit [35] as well as patients hospitalized in internal

medicine wards [17]. However, in the present study, we observed that there was no significant difference in CRP levels between the deceased and survival groups.

The normal range of serum inorganic phosphorus is 2.5–4.5 mg/dL. In this study, the median serum inorganic phosphorus value of the deceased groups was 4.85 mg/dL. In contrast, the median serum inorganic phosphorus value of the survival group was 3.15 mg/dL, which was lower than the upper limit of the normal range. This difference in serum inorganic phosphorus between the two groups might affect a very-early prognosis. It has been reported that a serum inorganic phosphorus level higher than 5.5 mg/dL is associated with a further increase in the risk of cardiovascular and all-cause mortality for patients with mild or moderate renal function impairment [36]. Dhingra et al. reported a strong relationship between all-cause mortality for 45 months in patients with chronic kidney disease and serum inorganic phosphorus concentration [37]. Moreover, high serum inorganic phosphorus levels have been shown to be associated with the risk of cardiovascular disease and high mortality [37]. Naffa et al. have reported that elevated serum inorganic phosphorus levels affect the mortality rate of patients community-acquired pneumonia [38]. Another study in our hospital, targeting patients with high AST, also suggested that serum inorganic phosphorus might be an excellent variable to predict short-term prognosis [39]. Consistent with the above findings, our results indicate that serum inorganic phosphorus is associated with various kinds of disease and might be a noteworthy variable to predict short-term prognosis.

The BUN levels of the deceased groups were higher than those of the survival groups. Liu et al. reported that BUN was an independent predictor of in-hospital mortality in groups of patients with acute aortic dissection [40]. Miura et al. reported that a BUN increase during hospitalization was associated with all-cause death in the group of patients with acute heart

failure syndrome [41]. In acute ischemic stroke patients, increased BUN at admission was a significant prognostic factor associated with in-hospital mortality [42]. Furthermore, a recent report indicates that a high level of BUN is a significant predictor for in-hospital mortality in patients with acute myocardial infarction [43]. Moreover, increased BUN was robustly associated with adverse outcomes in critically ill patients admitted to an ICU, and associated with mortality in heart failure and renal failure patients [44]. Interestingly, a recent study supports that BUN levels are an independent predictor of long-term mortality in older patients [45]. BUN is associated with hospital mortality, and a combination of BUN and clinical signs might constitute a powerful but easy-to-use tool for risk stratification in critically ill patients and help to improve their outcome [46].

Limitations and future research

The current study has several limitations. First, this was a single-center retrospective study conducted at Saitama Medical Center in Japan, and our findings may not be generalizable to other populations. Second, we selected patients with CRP levels over 40 mg/dL, and the risk factors for 72-hour mortality might be different in people with elevated CRP levels that are lower than that threshold. Finally, our analysis was powered to detect a strong association between risk factors and outcome with a small OR < 4, which may have introduced false-positive results.

Conclusion and future research

Taken together, we found that the prognosis of patients with an extremely high level of CRP over 40 mg/dL is affected by age, higher serum inorganic phosphorus, and higher BUN. Our findings provide clinical evidence that when treating patients with an extremely high level of CRP, physicians can easily estimate the 72-hour mortality probability using age, serum inorganic phosphorus, and BUN to make a prediction and facilitate an easier decision-making process. However, the pathophysiological mechanism between 72-hour mortality of an extremely high level of CRP and the association with age, serum inorganic phosphorus, and BUN remains unknown. Further prospective studies are warranted to validate the performance of the mortality prediction model in patients with an extremely high level of CRP. Moreover, the mechanistic relationship between mortality in patients with extremely high CRP and older age elevated serum inorganic phosphorus, and BUN levels need to be identified. Overall, our findings provide clinical support to improve the quality of initial medical management for patients with extremely high levels of CRP over 40 mg/dL.

Acknowledgement

The summary of this study was presented at the Beginning of Medical Students and Residents for the Japanese Society of Internal Medicine in the 116th Annual Meeting of the Japanese Society of Internal Medicine (Nagoya, April 27, 2019). Editorial support, in the form of medical writing, assembling tables, and creating high-resolution images based on the authors' detailed directions, collating author comments, copyediting, fact-checking, and referencing, was provided by Editage, Cactus Communications, and funded by JSPS KAKENHI (Grant Number: 26460916).

Conflicts of interest

The authors state that they have no conflicts of interest.

Ethical approval/informed consent

The study protocol was approved by the Institutional Clinical Research Ethics Review Board of Saitama Medical Center, Jichi Medical University, Saitama, Japan (Clinical #10-79).

Funding

This work was supported by JSPS KAKENHI (Grant Number: 26460916). Hitoshi Sugawara received this grant award. The funders had no role in study design, data collection and analysis, decision to publish, or preparation of the manuscript.

Author contributions

Conceptualization: Kai Saito and Hitoshi Sugawara

Data curation: Kai Saito and Hitoshi Sugawara

Formal analysis: Kai Saito and Hitoshi Sugawara

Funding acquisition: Hitoshi Sugawara

Investigation: Kai Saito and Hitoshi Sugawara

Supervision: Akira Ishii, Tamami Watanabe, Takahiko Fukuchi and Hitoshi Sugawara

Writing - original draft preparation: Kai Saito and Hitoshi Sugawara

Writing - review, and editing: Akira Ishii, Tamami Watanabe, Takahiko Fukuchi and Hitoshi Sugawara

REFERENCES

1. Meystre SM, Lovis C, Burkle T, Tognola G, Budrionis A, Lehmann CU. Clinical Data Reuse or Secondary Use: Current Status and Potential Future Progress. Yearb Med Inform. 2017;26(1): 38-52.

2. Lucero RJ, Kearney J, Cortes Y, Arcia A, Appelbaum P, Fernandez RL, et al. Benefits and Risks in Secondary Use of Digitized Clinical Data: Views of Community Members Living in a Predominantly Ethnic Minority Urban Neighborhood. AJOB Empir Bioeth. 2015;6(2): 12-22.

3. Lundberg GD. Critical (panic) value notification: an established laboratory practice policy (parameter). JAMA. 1990;263(5): 709.

4. Mo SH, Yamazaki H, Koike T, Amemiya N, Endo T, Kazama F, et al. Proposal of establishing the critical values based on distributions of emergency test data. Jpn J Clin Lab Autom. 2001;26: 166–169.

5. Chapman CN, Otis CN. From critical values to critical diagnoses: a review with an emphasis on cytopathology. Cancer Cytopathol. 2011;119(3): 148-157.

6. Black S, Kushner I, Samols D. C-reactive Protein. J Biol Chem. 2004;279(47): 48487-48490.

7. Sproston NR, Ashworth JJ. Role of C-Reactive Protein at Sites of Inflammation and Infection. Front Immunol. 2018;9: 754.

8. Boncler M, Wu Y, Watala C. The Multiple Faces of C-Reactive Protein-Physiological and Pathophysiological Implications in Cardiovascular Disease. Molecules. 2019;24(11). pii. E2062. doi: 10.3390/molecules24112062.

9. Pepys MB, Hirschfield GM. C-reactive protein: a critical update. J Clin Invest. 2003;111(12): 1805-1812.

10. Kaur M. C-reactive Protein: A Prognostic Indicator. Int J Appl Basic Med Res. 2017;7(2): 83-84.

11. Cai Z, He W, Zhuang FJ, Chen Y. The role of high high-sensitivity C-reactive protein levels at admission on poor prognosis after acute ischemic stroke. Int J Neurosci. 2019;129(5): 423-429.

12. Zhou L, Cai X, Liu Q, Jian ZY, Li H, Wang KJ. Prognostic Role of C-Reactive Protein In Urological Cancers: A Meta-Analysis. Sci Rep. 2015;5: 12733.

13. Xiao X, Wang S, Long G. C-reactive protein is a significant predictor of improved survival in patients with advanced non-small cell lung cancer. Medicine (Baltimore). 2019;98(26): e16238. doi: 10.1097/MD.0000000000016238.

14. Mager A, Hammer Y, Ofek H, Kedmi I, Iakobishvili Z, Kornowski R. Prognostic and Diagnostic Significance of Serum High-Sensitivity C-Reactive Protein Level in Patients with Acute Idiopathic Pericarditis. Isr Med Assoc J. 2019;21(11): 747-751.

15. Abolhassani AR, Schuler G, Kirchberger MC, Heinzerling L. C-reactive protein as an early marker of immune-related adverse events. J Cancer Res Clin Oncol. 2019;145(10): 2625-2631.

16. Sezgin Y, Bulut S, Bozalioglu S, Sezgin A. Levels of High-Sensitivity C-Reactive Protein in Heart Transplant Patients With and Without Periodontitis. Exp Clin Transplant. 2019;17(Suppl 1): 123-127.

17. Goltzman G PS, Cohen L, Avivi E, Rapoport MJ. Single Admission C-reactive protein Levels as a Sole Predictor of Patient Flow and Clinical Course in a General Internal Medicine Department. Isr Med Assoc J. 2019;21: 686–691.

18. Miller M, Zhan M, Havas S. High attributable risk of elevated C-reactive protein level to conventional coronary heart disease risk factors: the Third National Health and Nutrition Examination Survey. Arch Intern Med. 2005;165(18): 2063206-8.

19. Vigushin DM, Pepys MB, Hawkins PN. Metabolic and scintigraphic studies of radioiodinated human C-reactive protein in health and disease. J Clin Invest. 1993;91(4): 1351-1357.

20. Shine B, de Beer FC, Pepys MB. Solid phase radioimmunoassays for human C-reactive protein. Clin Chim Acta. 1981;117(1): 13-23.

21. World Medical A. World Medical Association Declaration of Helsinki: ethical principles for medical research involving human subjects. JAMA. 2013;310(20): 2191-2194.

22. Silvestre J, Povoa P, Coelho L, Almeida E, Moreira P, Fernandes A, et al. Is C-reactive protein a good prognostic marker in septic patients? Intensive Care Med. 2009;35(5): 909-913.

23. Shell W, Peter T, Mickle D, Forrester JS, Swan HJ. Prognostic implications of reduction of left ventricular filling pressure in early transmural acute myocardial infarction. Am Heart J. 1981;102(3 Pt 1): 335-340.

24. Siregar NN SP, Subekti I, Muhadi M. . Seventy-Two Hour Mortality Prediction Model in Patients with Diabetic Ketoacidosis: A Retrospective Cohort Study. J ASEAN Fed Endocr Soc. 2018;33: 124–129.

25. Quan H, Li B, Couris CM, Fushimi K, Graham P, Hider P, et al. Updating and validating the Charlson comorbidity index and score for risk adjustment in hospital discharge abstracts using data from 6 countries. Am J Epidemiol. 2011;173(6) :676-682.

26. Faul F, Erdfelder E, Buchner A, Lang AG. Statistical power analyses using G*Power 3.1: tests for correlation and regression analyses. Behav Res Methods. 2009;41(4): 1149-1160.

27. Henje Blom E, Olsson EM, Serlachius E, Ericson M, Ingvar M. Heart rate variability is related to self-reported physical activity in a healthy adolescent population. Eur J Appl Physiol. 2009;106(6): 877-883.

28. Leon DA, Jdanov DA, Shkolnikov VM. Trends in life expectancy and age-specific mortality in England and Wales, 1970-2016, in comparison with a set of 22 high-income countries: an analysis of vital statistics data. The Lancet Public health. 2019;4: e575-e82. doi: 10.1016/S2468-2667(19)30177-X.

29. Olsen MH, Hansen TW, Christensen MK, Gustafsson F, Rasmussen S, Wachtell K, et al. Cardiovascular risk prediction by N-terminal pro brain natriuretic peptide and high sensitivity C-reactive protein is affected by age and sex. Journal of hypertension. 2008;26: 26-34.

30. Ryu JA, Yang JH, Lee D, Park CM, Suh GY, Jeon K, et al. Clinical Usefulness of Procalcitonin and C-Reactive Protein as Outcome Predictors in Critically Ill Patients with Severe Sepsis and Septic Shock. PLoS One. 2015;10(9): e0138150. doi: 10.1371/journal.pone.0138150.

31. Hajek V, Pasquet F, Karkowski L, Lachenal F, Gerome P, Pavic M. [Etiology and prognosis of highly elevated C-reactive protein levels (>/=500 mg/L): a retrospective study about 168 measures in a series of 113 patients]. Rev Med Interne. 2011;32(11): 663-668.

32. Toss H, Lindahl B, Siegbahn A, Wallentin L. Prognostic influence of increased fibrinogen and C-reactive protein levels in unstable coronary artery disease. FRISC Study Group. Fragmin during Instability in Coronary Artery Disease. Circulation. 1997;96(12): 4204-4210.

33. Shrotriya S, Walsh D, Nowacki AS, Lorton C, Aktas A, Hullihen B, et al. Serum C-reactive protein is an important and powerful prognostic biomarker in most adult solid tumors. PLoS One. 2018;13(8): e0202555. doi: 10.1371/journal.pone.0202555.

34. Schmid M, Schneitter A, Hinterberger S, Seeber J, Reinthaller A, Hefler L. Association of elevated C-reactive protein levels with an impaired prognosis in patients with surgically treated endometrial cancer. Obstet Gynecol. 2007;110(6): 1231-1236..

35. Karasahin O, Tasar PT, Timur O, Yildirim F, Binici DN, Sahin S. The value of C-reactive protein in infection diagnosis and prognosis in elderly patients. Aging Clin Exp Res. 2018;30(6): 555-562.

36. Suki WN, Moore LW. Phosphorus Regulation in Chronic Kidney Disease. Methodist Debakey Cardiovasc J. 2016;12(4 Suppl): 6-9.

37. Dhingra R, Sullivan LM, Fox CS, Wang TJ, D'Agostino RB, Sr., Gaziano JM, et al. Relations of serum phosphorus and calcium levels to the incidence of cardiovascular disease in the community. Arch Intern Med. 2007;167(9): 879-885.

38. Naffaa ME, Mustafa M, Azzam M, Nasser R, Andria N, Azzam ZS, et al. Serum inorganic phosphorus levels predict 30-day mortality in patients with community acquired pneumonia. BMC Infect Dis. 2015;15: 332.

39. Saito K FT, Watanabe T, Sugawara H, editor Creation of a novel 72-hour outcome prognosis prediction model for patients with high critical levels of aspartic aminotransferase: Cross-sectional study, Case-control design. Proceeding of American College of Physicians Japan Chapter, 2019; Kyoto, Japan.

40. Liu J, Sun LL, Wang J, Ji G. Blood urea nitrogen in the prediction of in-hospital mortality of patients with acute aortic dissection. Cardiol J. 2018;25(3): 371-376.

41. Miura M, Sakata Y, Nochioka K, Takahashi J, Takada T, Miyata S, et al. Prognostic impact of blood urea nitrogen changes during hospitalization in patients with acute heart failure syndrome. Circ J. 2013;77(5): 1221-1228.

42. You S, Zheng D, Zhong C, Wang X, Tang W, Sheng L, et al. Prognostic Significance of Blood Urea Nitrogen in Acute Ischemic Stroke. Circ J. 2018;82(2): 572-578.

43. Horiuchi Y, Aoki J, Tanabe K, Nakao K, Ozaki Y, Kimura K, et al. A High Level of Blood Urea Nitrogen Is a Significant Predictor for In-hospital Mortality in Patients with Acute Myocardial Infarction. Int Heart J. 2018;59(2): 263-271.

44. Arihan O, Wernly B, Lichtenauer M, Franz M, Kabisch B, Muessig J, et al. Blood Urea Nitrogen (BUN) is independently associated with mortality in critically ill patients admitted to ICU. PLoS One. 2018;13(1): e0191697. doi: 10.1371/journal.pone.0191697.

45. Sullivan DH, Sullivan SC, Bopp MM, Roberson PK, Lensing SY. BUN as an Independent Predictor of Post-Hospital-Discharge Mortality among Older Veterans. J Nutr Health Aging. 2018;22(7): 759-765.

46. Wernly B, Lichtenauer M, Vellinga NAR, Boerma EC, Ince C, Kelm M, et al. Blood urea nitrogen (BUN) independently predicts mortality in critically ill patients admitted to ICU: A multicenter study. Clin Hemorheol Microcirc. 2018;69(1-2): 123-131.

Figure 1

Click here to download Figure Fig1.tif

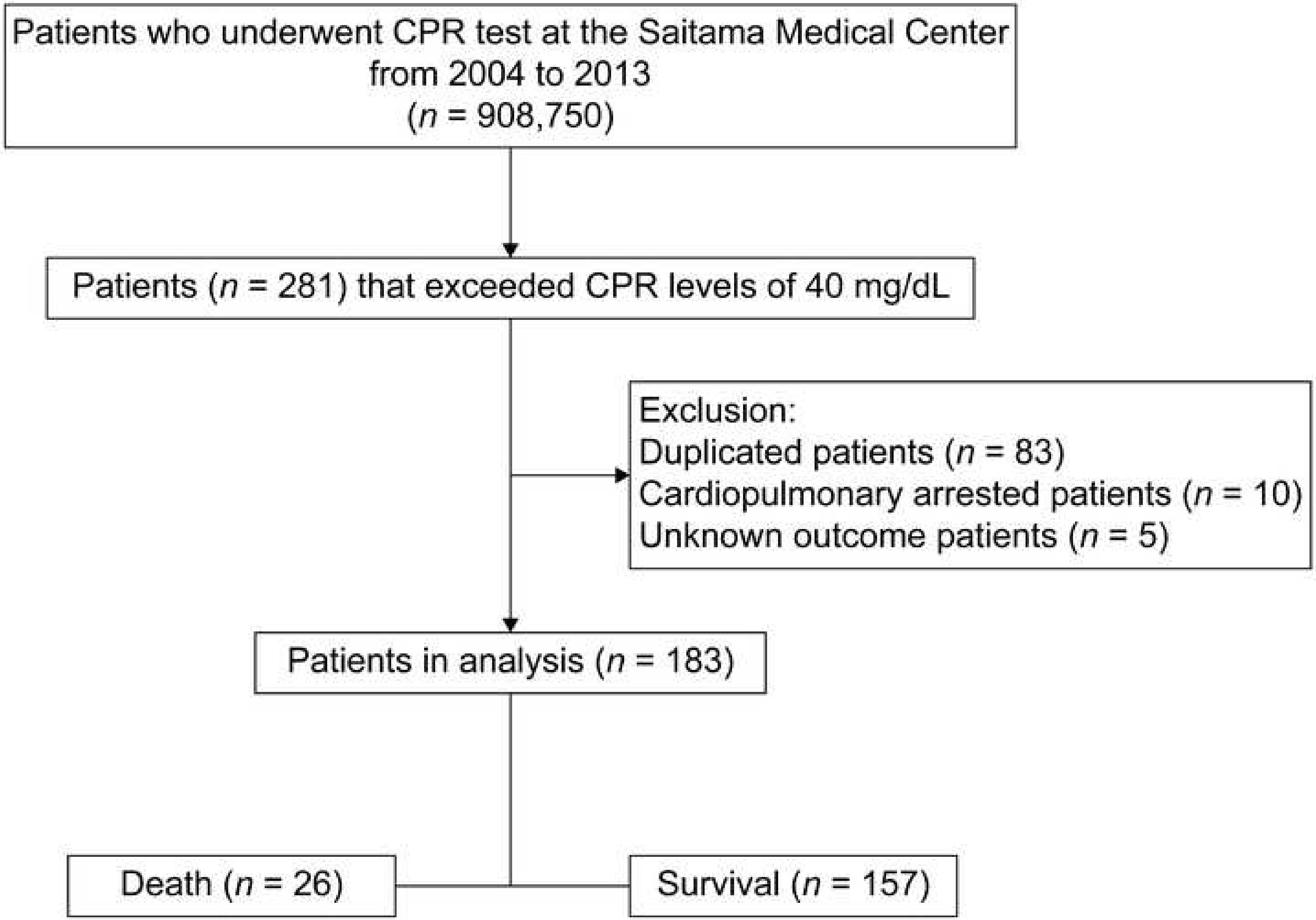

Figure 2

Click here to download Figure Fig2.tif

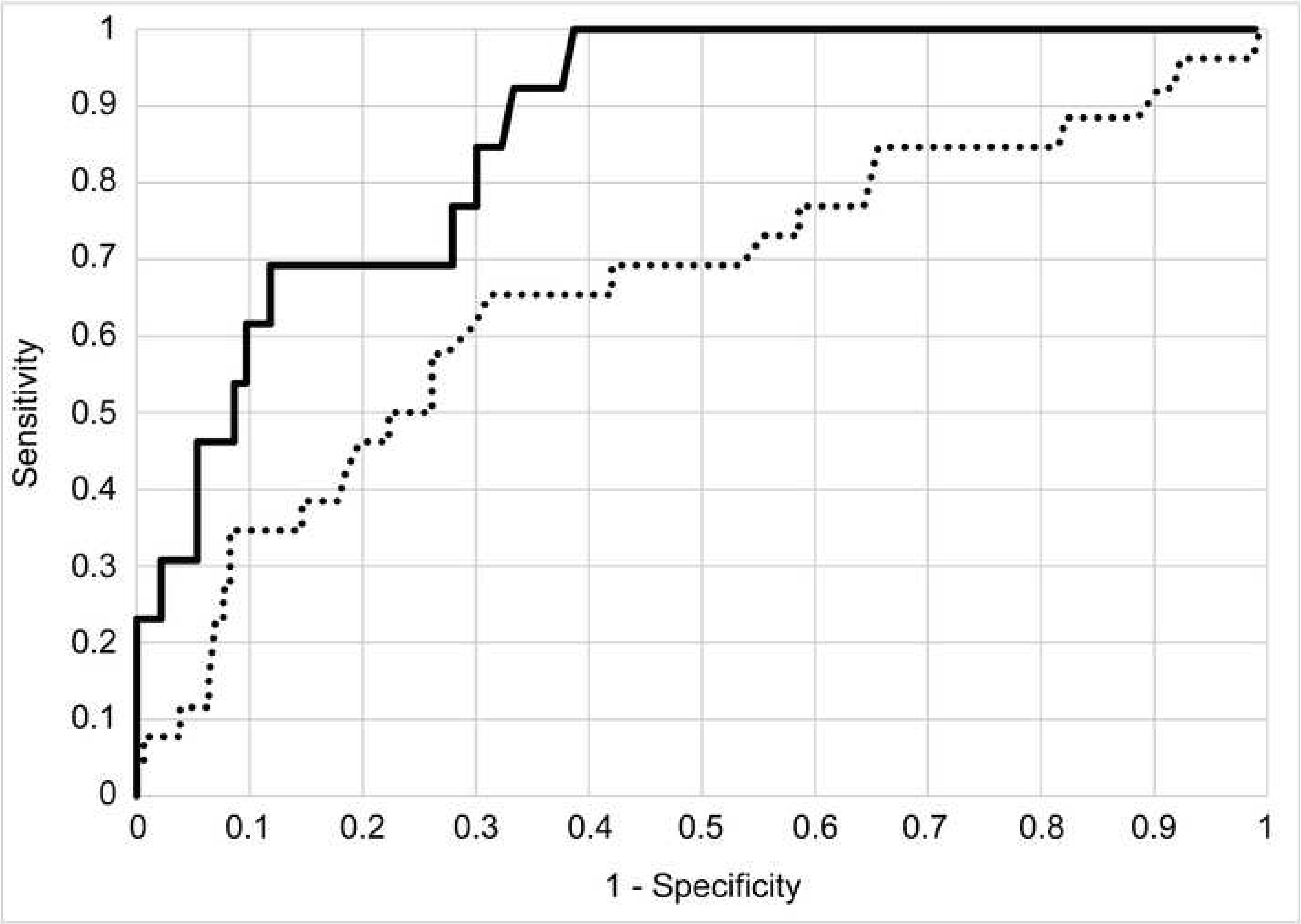

Supplement: Attachment — Submitted filename: PONE-D-20-07042_reviewer_PLOS notes.pdf [file pone.0246259.s005.pdf]
